# Does introduction of a Patient Data Management System (PDMS) improve the financial situation of an intensive care unit?

**DOI:** 10.1186/1472-6947-13-107

**Published:** 2013-09-16

**Authors:** Ixchel Castellanos, Jürgen Schüttler, Hans-Ulrich Prokosch, Thomas Bürkle

**Affiliations:** 1 Anästhesiologische Klinik, Universitätsklinikum Erlangen, Erlangen, Germany; 2 Lehrstuhl für Medizinische Informatik, Friedrich-Alexander Universität Erlangen-Nürnberg, Erlangen, Germany

**Keywords:** PDMS, CPOE, Return of investment, Costs, Intensive care unit

## Abstract

**Background:**

Patient Data Management Systems (PDMS) support clinical documentation at the bedside and have demonstrated effects on completeness of patient charting and the time spent on documentation. These systems are costly and raise the question if such a major investment pays off. We tried to answer the following questions: How do costs and revenues of an intensive care unit develop before and after introduction of a PDMS? Can higher revenues be obtained with improved PDMS documentation? Can we present cost savings attributable to the PDMS?

**Methods:**

Retrospective analysis of cost and reimbursement data of a 25 bed Intensive Care Unit at a German University Hospital, three years before (2004–2006) and three years after (2007–2009) PDMS implementation.

**Results:**

Costs and revenues increased continuously over the years. The profit of the investigated ICU was fluctuating over the years and seemingly depending on other factors as well. We found a small increase in profit in the year after the introduction of the PDMS, but not in the following years. Profit per case peaked at 1039 € in 2007, but dropped subsequently to 639 € per case. We found no clear evidence for cost savings after the PDMS introduction. Our cautious calculation did not consider additional labour costs for IT staff needed for system maintenance.

**Conclusions:**

The introduction of a PDMS has probably minimal or no effect on reimbursement. In our case the observed increase in profit was too small to amortize the total investment for PDMS implementation.

This may add some counterweight to the literature, where expectations for tools such as the PDMS can be quite unreasonable.

## Background

### Scientific background

Patient Data Management Systems (PDMS) support bedside clinical documentation at intensive care units (ICUs). They comprise components of computerized physician order entry (CPOE) such as daily drug and treatment orders. PDMS implementation costs between 15.000 and 20.000 Euros per bed [1] raise the question if these systems are cost effective.

### Rationale for the study

Costing for inpatient care in Germany (as well as in other countries) is based on Diagnosis Related Groups (DRG). Grouping patients into ICU-specific DRGs for reimbursement requires hospitals to document ventilation time, daily modified SAPS II and TISS Score, as well as several more parameters [2]. Missing or erroneous coding information leads to loss of revenues. In this context, Fränkel et al. detected that errors in the paper–based ventilation time documentation caused 22.7% false patient groupings in six of seven observed ICUs and reported an enormous negative cost effect [3]. Since our PDMS comes with a direct interface for automated ventilation time documentation, we assume that a reduction of missing or erroneous documentation could lead to visible economic effects, and potentially a return of investment (ROI) that justifies the implementation costs. Scanning the literature however, we only found direct cost and revenue calculations for RIS/PACS in radiology [4] but none for PDMS in ICU.

### Objectives of the study

To investigate the mentioned aspects, we performed a retrospective observational review of the development of costs and revenues at one of our ICUs to assess the potential cost benefit of the PDMS:

1. How do costs and revenues of an ICU develop before and after introduction of a PDMS?

2. Can higher revenues be obtained with improved PDMS documentation?

3. Can we present cost savings attributable to the PDMS?

### Study context

#### Organizational setting

Erlangen University Hospital, a tertiary care facility in southern Germany with 1320 beds, introduced a PDMS [5]. This study concerns a 25-bed interdisciplinary surgical ICU, where approximately 2500 patients are treated per year, with an average length of stay (LOS) of 3.3 days. A recovery room with 10 beds is attached.

#### System details and system in use

Beginning in October 2006, a commercial PDMS was introduced stepwise in each ICU subarea to replace the former paper based patient chart. This rollout was completed in December, so we consider 2007 as the first year with a fully operational PDMS in use. In 2007 and 2009 the PDMS was rolled out to five additional recovery room beds each year.

From January 2007, staff from all professional groups participated continuously in digital bedside charting of all patient activities. The PDMS complies with most requirements given in [6], and supports data reuse and data transfer for accounting purposes to the administrative subsystem. System functions comprise online data acquisition of vital signs from monitoring devices, from ventilators and from other medical devices. The PDMS supports bedside CPOE for orders between physicians and nursing staff and facilitates calculations of drug doses and fluid balances [7]. Import interfaces have been established for laboratory, microbiology and radiology data, as well as surgery reports. The PDMS supports automated calculation of ventilation times, automated scoring and semi-automated coding of diagnoses and procedures from previously documented clinical data according to the coding rules of the German DRG system. Furthermore, the PDMS offers a specific DRG workplace functionality to help the physician collect all data that is relevant for coding. The DRG workplace includes an interface for exporting DRG data directly to the electronic billing system of the hospital, thus avoiding potential transcribing errors. Various reporting functionalities, views in tabular or graphical format, printout functionalities and support for digital photography documentation are available. Some Arden syntax based decision support functions for low glucose alerting, administrative overviews and calculation of scores have been added by our workgroup [8].

## Methods

### Study design

We performed a retrospective observational and explorative analysis of available cost and reimbursement data of this ICU for a time period of three years before and three years after system introduction.

### Theoretical background

ROI calculations have been published for various electronic applications in national or international settings, including outpatient electronic records [9], CPOE systems [10] and clinical pathways [11]. These calculations, however, are often based on assumptions and estimations derived from small study populations with extrapolation to a larger collective. We scanned the literature for “effects” and “cost-and-benefit-analysis” and could not find a single source that demonstrated cost savings for a PDMS and described a ROI calculation based on real costs and revenues. Searching for studies on the PDMS-associated benefits, we located some reports on time savings [12]. We then widened our search to “medical information systems” and “computerized physician order entry”, and found sources for radiology systems comparing film costs directly to the costs for digital picture archiving [4]. For CPOE systems some authors [10,13] calculate positive effects such as avoiding adverse drug events as a financial benefit. There is, however, no evidence that these revenues have in fact been realized [14].

### Participants

Billing data collected over six years from a 25 bed interdisciplinary surgical ICU. All data was obtained from the SAP billing system of the hospital.

### Study flow

Our intention was to obtain stabilized economic data free from short term fluctuations. The analysis was conducted retrospectively in 2011, considering uninterrupted data from 2004 to 2009. We included all cases with a stay at the investigated ICU. Patients staying overnight in the recovery room were likewise treated as ICU patients, and were thus also included in the analysis. The German DRG system was introduced in 2003, and its practical application became mandatory in 2004. In the following years the system underwent some modifications, bringing the number of DRGs from 806 (in 2004) to 1147 (in 2009). The PDMS, after its introduction in 2006, also underwent regular maintenance and one update per year, which included adaptation to the altered DRG coding rules.

### Outcome measures

The main outcome parameter was the increase or decrease in profit for the ICU in the year after introduction of the PDMS. Profit is calculated as the difference between reimbursements and costs for patient care at the ICU, but should not be misinterpreted as economic net profit. ICUs are part of a complex clinical environment with reciprocal financing between departments and units. In our analysis, profit is rather seen as a more general parameter for measuring and visualizing changes in the economic processes over the years. These numbers were compared to required PDMS investment costs and their amortization. We included the number of cases, the DRG-based case mix index, LOS, and the total count of nursing days as reference parameters.

### Methods for data acquisition

We followed the ten point checklist of Drummond [15] for economic evaluation of healthcare programs except for the prospective trial design. Cost data was derived from the hospitals central accounting software using the standardized annual cost reports of the ICU cost unit. Reimbursement data was extracted from standardized monthly controlling reports for the same cost unit. For reference we also included the German annual inflation rate available from the German Federal Statistics Bureau.

Costs for the PDMS implementation were split into investment costs and yearly running costs. Investment costs are covered by a hospital-wide PDMS implementation budget and derived from a separate PDMS investment plan for university hospitals with funding from the German government. Yearly running costs are charged directly to the ICU and derived from the ICU cost unit data. Running costs before the PDMS introduction comprise maintenance charges for the workstations, as well as costs for some specific data processing applications in the ICU. After PDMS implementation, current data processing costs include the ICU’s share in maintenance charges payable to the PDMS and database vendor. Labour costs for clinical and IT staff in the pre-implementation configuration period have been calculated on basis of allocated working time, and are included in the investment costs [5]. A person year was calculated with 220 person days at € 40.000 for nursing staff, and at € 60.000 for physicians and IT staff. Costs for additional centralized IT staff required to maintain the PDMS on meanwhile eight ICUs have not been included.

### Methods for data analysis

The average LOS was computed dividing the number of ICU days by the number of patients in this period. The case mix index (CMI) in the German DRG system is calculated by dividing the sum of all cost weights of the underlying cases (the case mix) by the number of cases, and can be used as an indirect parameter to describe the average case severity. All data analysis is descriptive due to the explorative study design.

## Results

### Demographic and other study coverage data

The essential demographic and cost data is displayed in Table 1. The number of treated patients (DRG cases) increased from 1,853 to 2,190, associated with a drop of the average ICU LOS from nearly four to approximately three days. CMI rises from 4.95 in 2004 to a maximum of 6.3 in 2007 dropping back to 5.89 in 2009. The total number of nursing days fluctuates between 6,830 and 7,365 per year. In comparison with other countries, the German inflation rate is fairly low between 0.3 and 2.6%.

**Table 1:** Total costs and revenues of ICU cost unit

	**2004**	**2005**	**2006**	**2007**	**2008**	**2009**
No. cases	1,853	1,744	1,670	1,662	2,056	2,190
CaseMixIndex	4.95	5.27	5.80	6.30	5.79	5.89
Length of stay [days]	3.7	3.6	3.8	3.6	3.1	3.0
Total nursing days	7,365	6,956	7,102	6,830	6,946	7,022
Total ICU costs	8,938,352 €	8,910,418 €	9,212,039 €	9,360,012 €	10,129,576 €	10,583,335 €
Total ICU revenue	8,275,542 €	9,348,546 €	10,810,164 €	11,087,202 €	11,444,147€	12,270,127€
Profit	−662,810 €	438,128 €	1,598,125 €	1,727,190 €	1,314,571 €	1,686,793 €
German inflation rate	1.6%	1.6%	1.5%	2.3%	2.6%	0.3%
Profit / case	−358 €	251 €	957 €	1039 €	639 €	770 €
Difference to baseline 2006				129,065 €	−283,544 €	88,668 €

### Unexpected events during the study

We did the same database queries for reimbursement data several times and noticed that we were unable to obtain reproducible results until at least two years after finishing an observation period. It proved extraordinarily complicated to calculate the true revenues of the ICU for patients who were later discharged from another surgical unit, because their reimbursements were not assigned to the ICU in the hospital’s billing system.

### Study findings and outcome data

Table 1 lists total costs and revenues for the investigated ICU. Total costs and revenues of the ICU increased from 8.9 million to 10.6 respectively 8.3 to 12.3 million Euros. Net profit increased from 1.598 million in 2006 to 1.727 million € in 2007. Thus, a difference of 129,065 € could potentially have been caused by the PDMS. In 2008, net profit was lower than in 2006 and rose again in 2009. Profit per case revealed a major gain in the years 2004 to 2006 (prior to PDMS) peaking at 1,039 € per case in 2007, followed by a drop to 639 € per case in 2008 and 770 € per case in 2009. This trend coincided with the development of the CMI described above.

Table 2 lists ICU data processing expenses incurred in the years 2004–2009. Investment costs were covered from the German government while current data processing costs must be compensated with revenues from patient care. The first row illustrates the investment costs for the 25 beds ICU incurred in 2006 and further investment costs for the ten bed recovery room which was later equipped with the PDMS.

**Table 2:** Data processing costs split in investment costs and current costs

	**2004**	**2005**	**2006**	**2007**	**2008**	**2009**
Investment costs			826,000 €	73,636 €		37,260 €
Current costs	23,765 €	16,081 €	23,068 €	70,136 €	80,459 €	54,482 €
Total costs	23,765 €	16,081 €	849,068 €	143,772 €	80,459 €	91,742 €

These costs soared to around € 80,000, but then fell back in 2009 when more ICUs of the hospital introduced the same PDMS, resulting in decreased costs per client. Note: Costs for additional IT staff in the central IT department for PDMS maintenance are not included, because these are not billed directly to the ICU.

Considering the total investment sum for PDMS implementation (936,896 €), the small profit gain does not permit amortization.

### Unexpected observations

We did not expect costs and revenues to increase as much as they did. Over six years costs grew by 16% and revenues by 33%, which is far above inflation rate. Variables such as patient numbers or the CMI (which increased from an average of 5.34 to 5.99), grew notably after 2007. This may help to identify confounding factors such as increased disease severity or changing ICU assignments. It is certainly remarkable that yearly revenues ranged between 662,810 € loss and 1,727,190 € net profit. The biggest rise to around 1.6 million € profit, however, emerged between 2005 and 2006, one year before PDMS introduction.

## Discussion

### Answer to study questions

To the best of our knowledge, we are the first to present a ROI calculation for a PDMS based on actual costs and revenues of an ICU. We found:

1. A considerable increase of costs and reimbursement over six years, which was largely unaffected by PDMS introduction.

2. A small increase of 129,065 € profit between the years 2006 and 2007 which could have been attributable to the PDMS.

3. No net cost savings when comparing the considerable investment costs with the mentioned potential effect on revenue.

Considering previous reports with 22.7% erroneous patient groupings due to faulty paper-based ventilation time documentation [3] the use of the PDMS has likely eliminated a majority of potential errors for this type of data. Ventilation time is measured automatically from values imported from the ventilator interface. Appropriate procedure codes are proposed based on this data in the DRG workplace. All ventilation periods plus codes are digitally transferred to the billing system. Nevertheless, this specific financial effect of PDMS introduction was marginal when compared to other effects such as the general increases in the CMI and costs and reimbursements. Profit per case increased by 82 € but was lower in all subsequent years, indicating that the PDMS probably did not create sustained financial impact. Assuming the full benefit of 129,065 € is due to the PDMS, the amortization of nearly one million Euro total investment would exceed six years and therefore was not calculated. Besides, our calculation omits costs for additional central IT staff required for support and maintenance of the PDMS. Extra IT staff is needed in most cases, because the PDMS is a critical IT application which requires high system availability.

### Strengths and weaknesses of the study

We present a straight-forward method that permits to exclude major monetary effects of an information system based on continuous analysis of cost and reimbursement data over several years before and after system introduction. Things become more complicated in situations when a major profit increase is found, because then it remains unclear to which degree it can be attributed to the system or confounding factors. To cover such outcomes we have included an (incomplete) set of reference parameters such as CMI and number of cases which can be used to calculate relative values or fractions such as profit per case. We note the presence of confounding factors in our study such as the strong increase in CMI, costs and reimbursement between 2004 and 2006. These observations may be attributable either to more complicated patient cases or to changes in the German DRG system which evolved in these years to reflect the higher expenses incurred on ICUs. Other potential confounding factors include changes in ICU organization, management and strategy, reflected in increasing numbers of cases and decreasing length of stay within the total observation period.

In our design we did not measure other potential system benefits such as avoidance of medical errors or reduction of adverse drug events. Therefore our conservative approach may underestimate the benefits of the new PDMS compared to [10,16-18].

### Results in relation to other studies

As mentioned, we were unable to detect any study measuring real costs and reimbursement before and after the deployment of a PDMS. We found a variety of publications dealing with introduction of other medical information systems such as PACS, CPOE or CDSS. It is remarkable that even those publications rarely report actual costs and revenues. Instead, many authors measure or estimate benefits of the system, such as time savings or prevented adverse drug effects, and only calculate the potential financial benefit by extrapolation [4,10,12,17]. This may result in over- or underestimation of the system benefits. PDMS-centered studies such as [10,17,18] tend to concentrate on other positive system effects, such as an avoidance of medical errors and a reduction of adverse drug events. Stürzlinger and colleagues [19] published a review on several health technology assessment studies centered on CPOE introduction and found that CPOE benefits remain questionable, which fits nicely with our results. In their review, many studies were reported to have methodological flaws.

### Meaning and generalizability of the study

The method presented here can be easily reproduced at other institutions and for other types of information systems, if a continuous database for cost and reimbursement data is available for several years before and after system introduction. Potential confounding factors such as the number of cases, disease severity, staffing and organizational changes should be recorded in parallel. A monetary effect of the system can most likely be excluded if no relevant increase in profit after system introduction is found.

### Unanswered and new questions

It remains an open question if publications such as [3] have overestimated the effect of improved documentation, or if positive system effects have potentially been compensated by other negative effects in 2007. For future studies the collection of the most appropriate confounding factors and the assessment of their calculatory effects is a demanding task to better differentiate between system effects and side effects. These parameters must be comparable among different institutions and, most essentially, they must be available in good quality for a period of time before (!) and after the system introduction. This limits the selection of measured confounding parameters strongly, precisely because many relevant parameters are difficult to obtain completely for all patients before electronic documentation is deployed. Similar to measured quality parameters or system effects, confounding factors can be grouped according to Donabedian into the categories structure, process and outcome parameters [20]. Economic evaluation concerns outcome in terms of decreased expenditure, increased reimbursement or a combination of both. Therefore outcome parameters should also be considered in future research. These include mortality, LOS and ventilator time. Confounding process parameters have to be included as well. We measured the number of cases and disease severity using the CMI, but this economic parameter is simultaneously influenced by the quality of coding and managerial decisions. Unfortunately, the same can be said about many other parameters measuring disease severity. In the future, it may be better to use clinical scoring systems such as APACHE II or SAPS II. Furthermore, it may be advisable to consider also staffing, the number of admitted/discharged patients per day and other similar parameters to assess undesired effects influencing the cost data.

Generally, it is difficult to obtain conclusive results in evaluation studies of clinical information systems where only one evaluation method is applied. Therefore, multi-method evaluations, which combine different aspects such as documentation quality or time measurements with the described cost data analysis, are desirable. A prospective study design would be required to confirm our findings.

## Conclusion

Economic validation of healthcare information systems is an on-going work. The introduction of a PDMS has probably marginal or no effects on reimbursement. In our case the observed increase in profit was too small to amortize the total investment. This may add some counterweight to the literature, where expectations for tools such as the PDMS can be quite unreasonable. More studies are needed and evaluation methods must be improved to demonstrate that often postulated positive PDMS effects have in fact been realized. Considering the evaluation methodology, we were astonished to find few standardized or typical parameters which may be directly compared between different institutions. We hope that our approach may prompt more institutions to publish comparable data.

### Human subjects protections

This was an economical study analyzing economic effects before and after PDMS implementation on an intensive care unit with the aim of calculating a return of investment time. No formal intervention was performed. No additional patient data was collected. In according with German Bavarian Hospital Law (BayKrg §27) observational studies which do not collect additional patient data beyond routine documentation may use anonymized patient documentation without additional patient consent.

## Competing interests

The Erlangen-Nuremberg University Chair for Medical Informatics received a grant from Dräger Medical AG & Co. KG (PDMS provider) for improving some PDMS functions in a development partnership.

## Authors’ contributions

Study conception and design: IC, TB. Acquisition of data: IC, TB. Analysis and interpretation of data: IC, TB, JS, HUP. Drafting of manuscript: IC, TB. Critical revision: IC, TB, HUP, JS. All authors read and approved the final manuscript.

## Pre-publication history

The pre-publication history for this paper can be accessed here:

http://www.biomedcentral.com/1472-6947/13/107/prepub
